# Moderate and Severe Level of Food Insecurity Is Associated with High Calorie-Dense Food Consumption of Filipino Households

**DOI:** 10.1155/2021/5513409

**Published:** 2021-11-03

**Authors:** Imelda Angeles-Agdeppa, Marvin B. Toledo, Jezreel Ann T. Zamora

**Affiliations:** Department of Science and Technology, Food and Nutrition Research Institute, Bicutan, Taguig City, Philippines

## Abstract

Food insecurity is often deeply rooted in poverty. Hence, accessibility and the quality of foods consumed may affect the dietary pattern. The study aims to assess the relationship between food insecurity and dietary consumption. This investigation analyzed the data from the 2015 Updating of Nutritional Nutrition Survey. The Household Food Insecurity Access Scale (HFIAS) was used to determine household food security status and the prevalence of food insecurity. Food weighing, food inventory, and food recall were the methods used to collect food consumption data of sampled households. The study revealed poor nutrient quality and a greater likelihood of inadequacy of nutrients among moderate and severe food insecure households. Mild, moderate, and severe levels of food insecurity were found to affect 12%, 32%, and 22% of the population, respectively. The test showed that both moderate and severe food insecure families have significantly lower mean consumption of meat, milk, and fats and oils in contrast to food secure households. In comparison with food secure households, moderate and severe food insecure households consume higher amounts of cereals and cereal products, rice, and vegetables. Moderate and severe food insecure households have higher consumption of total carbohydrates but have significantly lower average intake of vitamin A, riboflavin, niacin, and total fat related to food stable households. Moreover, the results of the multiple logistic regression revealed that food insecure households have a higher likelihood to be deficient in energy, protein, calcium, vitamin A, thiamin, riboflavin, niacin, and vitamin C intakes, but except for iron (*p* value <0.05). Indeed, household food insecurity was associated with the higher consumption of calorie-dense food among Filipino households. This explains a lower nutrient quality and a higher likelihood of inadequacy of nutrients among moderate and severe food insecure households.

## 1. Introduction

The Committee on World Food Security (CFS) stated that food security occurs when all people, throughout all times, have both physical and economic access to sufficient, safe, and nutritious meals to fulfill their dietary needs as well as their food preferences for them to live an active and healthy life [1]. Nutrition is a crucial human need, which is why lack of food will have major consequences, such as hunger, obesity, cancer, and poverty [2]. The UN FAO latest findings have shown that 9% of the world's population was severely food insecure, while 17% experienced moderate levels of food insecurity. Food insecurity impacts 26.4 percent or approximately 2 billion of the global population, especially both moderate and severe food insecurity levels [3]. In the Philippines in 2015, more than half of the Filipino families were suffering from moderate food insecurity (32%) and severe food insecurity (22%) [4].

Food insecurity may be associated with poor nutrition. Even so, the relationship between food insecurity and dietary patterns is not yet fully established considering the number of local studies regarding this matter. The amount, variation, or combination of different food items in a meal, as well as the frequency of consumption, is referred to as a dietary pattern [5]. Earlier studies have connected food insecurity with decreased consumption of healthy foods and poor dietary quality with specific reference to low fruit and vegetable consumption [6, 7]. In a previous study, among children, child food insecurity is associated with a lower vegetable intake and a greater calorie, fat, sugar, and fiber consumption [8]. Food insecurity was also found to be associated with lower HEI levels and increased consumption of added sugars as well as empty calories in a 2003–2010 NHANES study [9].

With increasing interest in the significance of food insecurity as a health factor, the number of studies examining the link between food insecurity and dietary patterns has increased significantly in recent years but no Philippine data are generated for use locally by program planners. The purpose of this study is to evaluate the relationship between food insecurity with dietary patterns and food sources of households in the Philippines.

## 2. Materials and Methods

### 2.1. Research Design and Study Population

The present study was derived from the 2015 Updating of National Nutrition Survey results which was carried out by the Food and Nutrition Research Institute of the Department of Science and Technology. This is a cross-sectional survey that utilized a stratified three-stage sampling approach to represent all 17 regions and 80 provinces across the country, with a coverage rate of 96.6 percentage in both urban areas and rural areas. The first stage involved choosing primary sampling units (PSUs), which were made up of one barangay (village) or a group of adjacent barangays with at least 500 households. Enumeration areas (EAs) were determined within each primary sampling unit as for the second stage. Each EA comprises 150 to 200 households situated in a contiguous area in a barangay. The third and final stage involved selecting households from the sampled EA. The selected households served as the ultimate sampling unit during this stage.

A total of 9,930 sampled households were selected for the study. However, about 262 homes were excluded due to missing data of the variables of interest on the database arriving at a sum of 9,668 households for the analysis in this study. The DOST-FNRI Institutional Ethics Research Committee (FIERC) authorized the data collection instruments and survey protocol utilized in this study (FIERC protocol code: FNRI-2015-006). All surveyed households signed an informed consent form before taking part in the study.

### 2.2. Data Collection

#### 2.2.1. Household Dietary Consumption

Researchers used a digital measuring scale (Sartorius AZ4101 Digital Dietary Balance) to weigh household food items. All food prepared and served to the household for the day was weighed before cooking or in its raw state. Plate squanders, given-out food, and leftover food were also weighed in order to determine the actual weight of the food consumed. Nonperishable items that could be used during the measuring day, such as coffee, sugar, salt, cooking oil, and various condiments, were weighed at the beginning and end of the day. Household food consumption was recorded in terms of kind and amount. The researchers validated food weighing by weighing similar food items consumed by the household members outside the home. A 24-hour food recall was conducted among household members via face-to-face interview, wherein household members were asked to recall their food consumption. Most of the time, food recalled was in a cooked state. Other foods which were eaten raw were reported in their raw state. To determine the size of various food items consumed, devices such as wooden matchboxes, tablespoons, and plastic circles were utilized.

Before analysis, four steps of data validation and assessment of acquired data were used: (1) the dataset was modified and verified to guarantee accurate and high-quality survey data, and each food item has a matching food ID code based on the Philippines Food Composition Table (PhilFCT); (2) edited food item data were encoded in the Household Dietary Evaluation System (HDES), a computer system that translates food items to energy and nutrient consumption per household; (3) the HDES transformed all food weights into gross weight or “as purchased weight,” which was formerly the standard unit of encoding food weights. The actual weight of food consumed each day was calculated as the gross food weight lesser than the combined weights of remaining and discharged food and plate wastes; (4) the energy and nutrient intakes of households were compared to the energy and nutrient requirements outlined in the Philippine Dietary Reference Intakes (PDRI). Energy consumption was contrasted to the Recommended Energy Intake (REI), whereas nutrient intake was matched to the estimated average requirement (EAR). The results were given as a proportion of families that did not achieve the recommended calorie intake.

To compute for the household food consumption, the raw intake of each food group/nutrient was divided by the consumption unit (CU). In this study, CU was calculated such that one member or a guest consumed all major meals for the whole day at home. However, it should be emphasized that per capita reporting of family food intake has several limitations because it does not account for age, gender, or physiological differences among household members.

Ten food groups were utilized in the research to explore the food pattern consumed in all families based on their level of food insecurity. All reported meals and drinks were assigned to one of the ten food categories (Table 1).

#### 2.2.2. Household Food Security

The Household Food Insecurity Access Scale (HFIAS), which is specifically a pretested questionnaire, was utilized in the present study to identify levels of food security among Filipino households. A licensed nutritionist-dietitian conducted the face-to-face interviews and administered the questionnaire to the study participants. The questions were based on the household's food intake during the previous month, followed by inquiries on how frequently the family unit encountered the circumstances. The HFIAS categorizes food insecurity into four levels: food secure, mild, moderate, and severe.

Table 2 categorizes the types of food insecurity faced by households based on their frequency level. A food secure household does not encounter any of the circumstances or only has to worry about food on rare occasions. A family becomes slightly food insecure if it is occasionally or frequently concerned about food and/or is unable to consume preferred meals and/or rarely has to eat fewer diverse foods and/or to eat foods they dislike. A moderately food insecure household sacrifices food quality by eating a less varied diet and/or undesirable foods on a regular or irregular basis and begins to reduce the number of foods by reducing the meal portion or the number of meals on a regular or irregular basis, but it does not experience the three most severe conditions. A severely food insecure household often decreases the amount of food consumed and exhibits the three most severe symptoms (running out of food, going to sleep, being hungry, and not eating for the whole day). Any family experiencing any of the three severe situations is already classified as highly food insecure [10].

#### 2.2.3. Food Consumption Score

The food consumption score (FCS) is a frequency-weighted diet variety score based on a household's frequency of consuming various categories of food in the last seven days before survey administration. The FCS was estimated based on the variety of family intake of nine food groups: major staples, vegetables, fruits, meat and fish, oils, sauces, sugar, milk, and pulses. These were weighted by quality of nutrients that it adds to the diet multiplied by the frequency (number of days) of intake (Table 3) [11].

Households with a score of less than 28 are deemed to have inadequate food intake, with 28 and 42 scores were considered as borderline food consumption, while scores over 42 were judged to have adequate food consumption (Table 4).

#### 2.2.4. Socioeconomic and Demographic Data

Data on family economic status (wealth status), household size, place of household residence, sex of the household head, educational level and occupation level of the family head, and other household profiles were collected in this survey. The wealth index of Filipino households was determined through principal component analysis (PCA) which was based on variables such as household characteristics, household assets, infrastructure factors, and utility access. Scores were designated to each of the household asset and then was used to categorize wealth quintiles as poorest, poorest, middle, rich, and richest. The in-depth methods of measurement and categorization were presented elsewhere [12].

### 2.3. Statistical Analysis

Stata 15 was used for all statistical analyses performed in this study (Stata Statistical Software, release 15, Stata Corporation 2017). Frequency and percentages were used to present the characteristics of Filipino households. Mean, standard deviation, median, 25^th^ percentile, and 95^th^ percentile of food and nutrient intakes of the households were estimated to show the distribution of consumption by the food security level. For dichotomous, ordinal, and nominal categorical data, as well as measurement data, chi-square tests were employed to examine the relationships between household variables and food security levels. Differences in household food and nutrient intakes were compared to food security levels using one-way analysis of variance (ANOVA). Food and nutrient intakes were transformed through natural logarithm function ln(*x*).

Food pattern was analyzed by comparing the distribution of intakes of 10 food groups by the food security level. The diet quality was assessed based on the FCS scoring and percentage contribution of each food group to the total energy intake. Percentage contribution was calculated by summing the total energy for each food group divided by the overall sum of energy from all food multiplied by 100.

To estimate the relationship between dietary intake and food security level while adjusting for confounders, linear regression analysis was used in the association analysis. Unstandardized beta coefficients and 95% confidence intervals were also presented. Logistic regression analysis was applied to determine the odds of food and nutrient inadequacies related food security levels. The odds ratios (OR) and 95% confidence intervals were also presented in this study. Moreover, all models were analyzed both with and without adjustment. Confounder variables were household size, place of residence, sex, education, the household head's occupation, wealth quintile, electricity status, and type of toilet facility. All analyses set the significance level *α* at 0.05. All analyses were accounted for the sampling weights to reflect nationally representative results.

## 3. Result

The study included a total of 9,668 Filipino households, with nearly equal representation from rural and urban areas. The majority of Filipino households (67%) was found to be food insecure, with 12 percent, 32 percent, and 22 percent being slightly, moderately, and severely food insecure, respectively. In terms of family size, more than half (63%) have less than or equal to five family members, while 36% have more than five. Most of the household's heads were males (79%) and the majority has reached the elementary level (39%) and high school level (35%). About 38% family heads have low-income occupations while 6% have no occupation. The proportion of households was similarly distributed across the wealth quintile. Only 9% of the households do not have electricity and 14% either do not have a toilet or are not water-sealed.

All socioeconomic and demographic characteristics of the households included in this analysis were found to be significantly associated to food security levels. Specifically, seven out of ten (70%) family with ≤5 members were food secure. More than half (57–61%) of the homes in rural areas were moderate and severe food insecure. Forty percent of food secure family was under the richest quintile. Thirty-five percent of severe food insecure houses were in the poorest quintile and 25% in poor quintile. Moreover, the study found that half (51%) of severely food insecure households have a family head with an elementary education level. Half of the food secure household have a family head with high-income occupations, while almost half (47%) severe food insecure have low-income occupation. Households with no electricity (18%) and no toilet/not water-sealed (27%) have a higher rate of severe food insecurity (Table 5).

### 3.1. Food and Nutrient Intake according to Food Security Status of the Households

Mostly consumed food were cereals, rice, vegetables, and meat with an average consumption of 1508 g, 1303 g, 528 g, and 708 g, respectively. On the other hand, the least consumed foods were dried beans, nuts and peas (34 g), fats and oils (58 g), starchy roots and tubers (59 g), and sweetened beverages (61 g) (Table 6).

Among food secure households, cereals, rice, and meat were consumed with an average of 1356 g, 1193 g, and 708 g followed by vegetables (528 g) and milk (198 g). On the other hand, in mild food insecure households, the mean household food consumption cereals, rice, and meat were 1504 g, 1302 g, and 751 g accompanied by vegetables (528 g) and milk (184 g). Commonly consumed food among moderate and severe food insecure households were cereals, rice, and meat with 1590 g, 1357 g, and 637 g among moderate food insecure and 1611 g, 1389 g, and 576 g for severe food insecure households. Milk (moderate: 132 g, severe: 121 g), starchy roots, and tubers (moderate: 58 g, severe: 74 g), sugary sweetened drinks (moderate: 58 g, severe: 52 g), fats and oils (moderate: 56 g, severe: 51 g), and dry beans, nuts, and seeds (moderate: 56 g, severe: 30 g) are the least eaten foods among moderate and severe food insecure household (Table 6).

Tests showed that the moderate and severe food insecure group consumes considerably less meat, milk, and fats and oils than the food secure family. Severe food insecure households were also found to have lower mean intake of fruits and sugary sweetened beverages than food secure households. Moreover, moderate and severe food insecure households spend more cereals and cereal products, rice, and vegetables than food secure homes (Table 6).

The ANOVA test revealed a significant mean difference in expenditure across all food groups by food security index (*p* value<0.05). Multiple comparison tests showed that both moderate and severe food insecure groups have significantly lower mean consumption of meat, milk, and fats and oils compared to the food secure group. Severely food insecure households have lower average intake of fruits and sugary sweetened beverages related to food secure houses. On the other hand, both moderate and severe food insecure houses have a higher utilization of cereals and cereal products, rice, and vegetables contrasted to food secure houses (Table 6).

Overall, the mean household intake of total energy, carbohydrates, protein, and fat were 7607 kcal, 1340 g, 228 g, and 146 g. Iron was 38 mg, 1650 mg for calcium, 1649 *µ*g for vitamin A, 3 mg for thiamin, 3 mg for riboflavin, 76 mg for niacin, and 182 mg for vitamin C (Table 7).

Moderately food insecure households had a higher mean calorie intake as reflected in the higher total carbohydrate, less vitamin A, riboflavin, niacin, and total fat intake as compared with food secure. In addition, severe food insecure family has considerably lower mean calcium and thiamin consumption than food secure homes (Table 7).

ANOVA test showed that there were significant mean nutrient intake differences by the level of food security (*p* value <0.05) except for iron and vitamin C intake. Moderately food insecure households have higher mean calories related to food secure families. Moderately and severely food insecure households had been found to have higher mean consumption of total carbohydrates, but they have a significantly lower mean consumption of vitamin A, riboflavin, niacin, and total fat compared to food secure households. Severely food insecure households have significantly lower mean intakes of calcium and thiamin compared to households that are food secure (Table 7).

**Figure 1:**
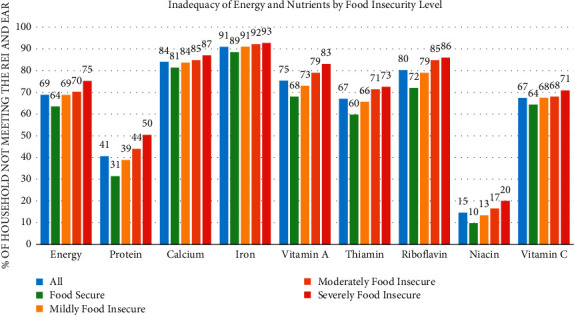
Proportion of households with inadequate energy and nutrient intake not meeting 100% REI and EAR intake by the food security level.

Results show that seven (7) out of ten (69%) households have met the 100% recommended energy intake (REI). The proportion of households not meeting the EAR's are protein (41%), calcium (84%), iron (91%), vitamin A (75%), thiamin (67%), riboflavin (80%), niacin (15%), and vitamin C (67%). The prevalence of inadequacy between energy and nutrients goes higher from food secure households to severely food insecure households. The Chi-square test showed a significant relationship between nutrient inadequacies and food security level at a 5% level of significance (Figure 1).

### 3.2. Food Security by Household Food Consumption Classification (FCS)

Almost half (49%) of the households with insufficient food consumption were severe food insecure, while 34% and 20% had borderline and acceptable consumption, respectively. On the other hand, the prevalence of food security was 36% for households with acceptable food consumption, 21% for borderline, and 16% for households with low food expenditure. The level of food consumption score appeared to have no large difference but had a small indication of the trend for moderately food insecure (26–35%) and mild food insecure families (9–13%). To support this statement, chi-square test confirmed that levels of food security were significantly associated with the FCS score categories at 5% level of significance (Table 8).

### 3.3. Food Security by Sources of Foods and Nutrients

Overall, about 68% of the household total energy consumption was from rice, 14% from meat, 7% from fats and oils, and the other percentages were from contributor food groups such as sweetened beverages, vegetables, milk, fruits, eggs, dried beans, nuts and seeds, and starchy roots and tubers. Rice remained the top 1 contributor of calorie expenditure of Filipino households across food security levels. However, about 74% of the energy intake of severely food insecure families was from rice, and it goes down to 71% for moderately food insecure, to 67% for mildly food insecure, and to 63% for food secure. Moreover, the contribution of meat to the household calorie consumption was 18% for food secure, and this is on a decreasing trend with food insecurity levels: mild, 14%; moderate, 12%; and severe, 10%. The contribution of fat across food security levels seems similar. The remaining groups such as fruits, vegetables, milk, eggs, and dried beans and nuts appear to have very low contributions to the caloric intake of the Filipino households (Table 9).

### 3.4. Regression Analysis

After adjustment for potential confounders such as household size, place of residence, sex, education and occupation of the family head, electricity status, and type of toilet facility and socioeconomic status, food security level was significantly associated with food consumption score (FCS) and nutrient intake of Filipino household except for total carbohydrates and vitamin A intakes. FCS decreased by −1.46 (95% CI: −2.45, −0.47) and −4.45 (−5.58, −10.63) for severely food insecure compared to food secure households. Total energy intake substantially declined by −331 (−535, −124.6) for severely food insecure in contrast to food secure households. Total protein intake reduced by −8 (−13.8, −2.17) for moderately food insecure and −18.5 (−25.1, −11.8) for severely food insecure in contrast to food secure homes. Total fat intake dropped by −11 (−18.7, −3.3) for severely food insecure. Calcium intake declined by −1.51 (−2.74, −2.1) for severely food insecure families. Iron intake fell by −72.9 (−142.5, −3.3) for severely food insecure households as compared to households that are food secure. Referring to food secure households, severely food insecure significantly decreased the consumption of thiamin by −0.21 (−0.35, −0.07). Riboflavin intake significantly declined by −0.14 (−0.25, −0.02) in the severe food insecure households. Niacin intake significantly decreased by −3.12 (−5.2, −1) and −5.9 (−8.3, −3.5) for moderately and severely food insecure households, respectively. Vitamin C intake diminished by −18.2 (−30.5, −6) for severely food insecure related to food secure households. Food security level appears to be not related to the change in the consumption of total carbohydrates and Vitamin A (Table 10).

Model 1 was adjusted to account for household size, place of residence, and sex of the household head. Results showed that the likelihoods of poor/borderline FCS increased by 3 times for severely food insecure compared to food secure families (95% CI: 3.14.25). The odds of poor/borderline FCS were approximately 2 times more likely among moderately food insecure related to food secure households (95% CI: 1.63–2.24). The probability of poor/borderline FCS inclined by 1.3 times for mildly food insecure than food secure homes (95% CI: 1–1.6). The prevalence of inadequate energy increased by 1.57 times among severely food insecure in comparison with food secure families (95% CI: 1.37–1.79). Moderately food insecure households were found to be 1.25 times more likely to have inadequate energy intake as compared to food secure households (95% CI: 1.12–1.37). Mildly food insecure household were 1.20 times more likely to be inadequate of energy compared to food secure household (95% CI: 1.03–1.4). The prevalence of protein inadequacy increased by 2 times among severely food insecure households correlated to food secure households (95% CI: 1.83–2.33). Moderately and mildly food insecure group were 1.61 and 1.33 times more likely to be inadequate of protein intake compared to food secure households, respectively. The odds of inadequate calcium intake inclined by 1.45 times among severe food insecure households in contrast to food secure households (95% CI: 1.23–1.71). Moderately food insecure families were 1.24 times more likely to have insufficient intake of calcium correlated with food secure houses (95% CI: 1.1–1.42). Inadequate intakes of iron were 1.43 (95% CI: 1.16–1.77) and 1.37 (95% CI: 1.14–1.64) times more expected for severe and moderate food insecure group in comparison with food secure households, respectively. The likelihoods of the inadequacy of vitamin A were 2.1 times more likely for severely food insecure (95% CI: 1.78–2.38), 1.6 times more likely for moderately food insecure (1.41–1.81), and 1.19 times more likely for mildly food insecure (95% CI: 1–1.4) compared to food secure households. The probability of inadequacy of thiamin was 1.64 times higher among severely food insecure (95% CI: 1.45–1.87), whereas for moderately and mildly food insecure, the odds increased by 1.56 (95% CI: 1.39–1.75) and 1.23 (95% CI: 1.1–1.42), respectively, compared to food secure homes. Severely food insecure group was 2.15 times more likely to be inadequate of riboflavin in connection to food secure families (95% CI: 1.82–2.51). Moderately and mildly food insecure households were 1.91 (95% C: 1.67–2.19) and 1.35 (95% CI: 1.14–1.61) times more expected to be deficient of riboflavin compared to food secure households. Severely, moderately, and mildly food insecure households were 2.15 (95% CI: 1.82–2.54), 1.70 (95% CI: 1.45–2), and 2.37 (95% CI: 1.11–1.69) times more probable to be insufficient of niacin correlated with food secure household, respectively. The test showed that the odds of having inadequate vitamin C intakes increased by 1.33 for severely food insecure households compared to households that are food secure (95% CI: 1.17–1.5). Moderately food insecure household was 2.28 times more feasible to be vitamin C impaired compared to food secure families (95% CI: 1.1–1.32) (Table 11).

Model 2 was adjusted for household size, educational level and occupation of the family head, place of residence, sex of the family head, electricity status, type of toilet facility, and socioeconomic status. The odds of poor FCS and nutrient deficiency seems to be equally likely for both mild food insecure and food secure households. Moderate food insecurity was 1.15 (95% CI: 1–1.30) times more prone to be inadequate of energy, 1.2 (95% CI: 1–1.34) times more expected to be inadequate of protein, 1.16 (95% CI: 1–1.34) times more prone to vitamin A deficiency, 1.19 (95% CI: 1.1–1.35) times more likely to be inadequate of thiamin, 1.34 (95% CI: 1.16–1.56) times more probably to have riboflavin deficiency, and 1.2 (95% CI: 1–1.43) times more likely to be insufficient of niacin. In comparison with food secure household, severely food insecure household was 1.69 (95% CI: 1.41–2) times more likely to have poor/borderline FCS, 1.45 (95% CI: 1.24–1.67) times more likely to be inadequate of energy, 1.43 (95% CI: 1.25–1.64) times more expected to be inadequate of protein, 1.26 (95% CI: 1.1–1.52) times more likely to be deficient of calcium, 1.38 (95% CI: 1.18–1.63) times more likely to be inadequate of vitamin A, 1.18 (95% CI: 1–1.36) times more possible to become inadequate of thiamin, 1.36 (95% CI: 1.24–1.63) times more likely to be insufficient of riboflavin, 1.35 (95% CI: 1.12–2.63) times more prone to niacin insufficiency, and 1.28 (95% C: 1.1–1.47) times more expected to be vitamin C deficient (Table 11).

## 4. Discussion

### 4.1. Food Group Consumption according to Food Security Status

The present study revealed that households that are moderately and severely food insecure were found to have higher mean consumption of cereals and cereal products, rice, vegetables, and starchy roots and tubers while having lower expenditure of fruits, meat, fish, and poultry, as well as milk and milk products, in comparison with food secure households. The results of the study were in conjunction with previous literature which found that food insecure families consumed more carbohydrate-rich foods [13, 14] and less animal source foods, protein-rich food, dairy products, and fruits [15, 16]. According to previous research, this could be attributed to the occurrence that, at lower income levels, households tend to consume more cereals as it is a cheap source of calories [17]. This is supported by a major hypothesis of previous research on food insecurity and diet, which suggests that food insecurity may result in a “substitution effect” where higher quality and/or less calorie-dense foods (including produce and lean sources of protein) are replaced with more energy-dense foods (often high in simple carbohydrates) that are less expensive as per-calorie basis [18]. Thus, given the lower cost of calorie-dense foods such as rice as well as starchy roots and tubers, food insecure households would more likely be incentivized to these, while consuming fewer amounts of nutrient-dense foods such as protein-rich foods that include meat, fish, poultry, and milk as well as fruits that are rich in micronutrients [19, 20]. Yet, on the contrary, the present study found higher consumption of vegetables which are nutrient-dense among food insecure households than the latter. Moreover, this study found a lower fat intake among food insecure households, which is different from the results of previous studies in western populations where it has been observed that food insecure households are more likely to consume high-fat foods due to a lack of resources [21–26], this could suggest the impact of geographical location on food consumption. In terms of the high consumption of rice, a possible explanation for this is that rice is a staple food for Filipinos. Thus, Filipinos living in moderate and severely food insecure households obtain their energy intake majorly from carbohydrates primarily rice rather than protein and fat sources. It is a dogma in the Philippines, especially among the lowest economic status households that rice supply connotes food security.

In terms of food group consumption and expenditures, an investigation regarding the relationship between food insecurity and overall daily capital (DPC) intake was conducted in a previous report which involved Bolivia, Burkina Faso, and the Philippines. The previous study found that, for food-secure households, the overall DPC food expenditure, as well as expenditure on animal goods, fruits, and fats and oils, was slightly greater (*p* = 0.05) in comparison with both households that are moderately and severely food insecure [27]. Poverty which is the common cause of food insecurity is stated in prior research to make consumers even more sensitive to changes in income and food prices because they do not have any safety nets in order to absorb income or price shocks when they purchase [28]. This is in line with the results of the 2018 eNNS in the Philippines, wherein the poorest households spent 42% of their total food purchases on energy-giving foods and 38% on body-building food, while the richest household spent more than half of their food purchase on body-building food which is more expensive and only spent 29% on energy-giving food [16]. Although in the present study, the association of food expenses to household food insecurity was not analyzed.

### 4.2. Food Consumption Score according to Food Security Status

Delving into one of the indicators of food security assessed in this study, food security is found to be significantly associated with food consumption score (FCS) (Figure 1). Moreover, a significant association was found between severe food insecure households and reduced food consumption scores. The present study revealed that almost half (49%) of severely food insecure households have poor food consumption, and an increased likelihood of poor food consumption scores (FCS <42%) is becoming more prevalent as the degree of household food insecurity worsens. Furthermore, poor food consumption was more pronounced in households that were moderately or severely food insecure.

A defining characteristic of food insecurity is limited or uncertain accessibility to sufficient food [29]. Moreover, according to a previous study, poor food consumption could be linked to the attributes of food insecure households characterized as those having a lower monthly per capita income, less desirable jobs, poor housing conditions, and lower levels of education, all of which can have an impact on their dietary intake [30]. These factors are stated by several previous studies to contribute towards poor/less food accessibility and availability [31, 32]. This could be also explained by the coping mechanism of food insecure households to poverty by reducing the quantity of food consumed to sustain their energy needs [33] or resorting to food shopping practices that are driven by efforts to reduce costs of food expenses [34] which could lead to poor food consumption.

### 4.3. Energy and Nutrient Intakes according to Food Security Status

Regarding nutrient intakes, household food insecurity, specifically, severely food insecure households, were found to be significantly associated with reduced consumption of total energy, total protein, total fat, calcium, iron, thiamin, riboflavin, niacin, and vitamin C except for total carbohydrates and vitamin A. Moreover, the higher severity of household food insecurity significantly increases the prevalence of inadequate total energy, total protein, calcium, vitamin A, thiamin, riboflavin, niacin, and vitamin C intake. Limited food accessibility, if prolonged, may explain the decline in nutrient intake observed among food insecure households and how it negatively affects nutritional status [35]. Indeed, the present study confirms findings of prior research, wherein it was suggested that household food insecurity is a marker of nutritional vulnerability which increases the susceptibility to nutrient inadequacies [14, 36, 37]. A previous study in Canada has shown compromised nutrient intake among food insecure households who are struggling with food sources [38]. In relation to the food group groups consumed, the reduced nutrient intakes on total protein, iron, and B vitamins may be ascribed to the lower consumption of meat, poultry, and milk products among food insecure households since these are major food sources for the nutrients aforementioned. The inadequate intake of fruits is also reflected in the reduced vitamin C intake among food insecure households. In a previous study, higher prevalence of deficiencies in nutrients such as protein, vitamin A, thiamin, riboflavin, vitamin B-6, folate, vitamin B-12, magnesium, phosphorus, and zinc are found among individuals living in food insecure households [39].

Thus, the present study suggests that food insecure households consume diets that are of poor nutrient quality which presupposes them to nutrient deficiencies. Inadequate nutrient intakes can adversely affect adults' [40, 41] and children's [37, 42, 43] health and well-being. This imposes the need for interventions targeting household food insecurity, particularly focusing on energy and nutrient intakes.

### 4.4. Contribution of Food Sources to Energy Intake according to Food Security Status

Pertaining to the percentage contribution of each food group to the total energy intake of Filipino households, rice remained as the major energy source regardless of the household food security level. Despite the fact that rice is the cheapest and most effective way to maintain a sustainable energy intake, it is considered nutritionally undesirable [44]. Moreover, rice-based diets are related to vitamin and iron deficiencies which in turn affect long-term food security [45]. The contribution of meat based on household calorie consumption was also found to be higher among food secure households (18%), and an alarmingly decreasing trend was observed with food insecurity levels: mild (14%), moderate (12%), and severe (10%). This is consistent with a previous study, which discovered that food secure households consume more meat than households that are food secure [46]. Meat could be also less consumed among food insecure households since it is more expensive than other food items [47]. The remaining food groups such as fruits, vegetables, milk, eggs, and dried beans and nuts appear to have very low contribution to the caloric consumption of both households that are food secure and food insecure (Table 8).

These findings connote that the present study may affirm previous literature which stated that when food is available, low-income households which suffer from food insecurity consume monotonous meals that are low in quality, cereal-based, and bereft of vegetables, fruit, and animal source foods, raising the risk of micronutrient deficiencies [48–50]. Monotonous diet which being reflected in the results of the contribution of food groups to energy intakes are found to be closely associated with food insecurity [49] resulting in malnutrition. Fruits and vegetables which were also found among the least consumed foods that contribute to a household's energy intake are also nutritionally beneficial since it is both rich in vitamins and minerals such as folate, vitamin A, vitamin C, and carotenoids [51] as well as dietary fiber and phytochemicals [52].

## 5. Conclusions

Household food insecurity was associated with dietary patterns among Filipinos. This is reflected in the higher consumption of calorie-dense foods among Filipino households experiencing moderate and severe food insecurity. This explains the results that lower nutrient quality and a higher likelihood of nutrient inadequacy or micronutrient deficiencies are observed in these households. Since food insecurity and dietary pattern are intertwined because both are economic issues, programs and policies addressing food insecurity in the Philippines may need to take steps to improve the whole context of the supply chain for products to be more available and accessible at a more affordable cost to improve quality and quantity of consumed food.

## Figures and Tables

**Table 1:** Classification of food groups.

Food groups	Example
Cereals and cereal products	Corn and corn products, other cereal products
Starchy roots and tubers	Cassava, potatoes, and sweet potatoes, other tubers
Rice and rice products	Rice and rice products such as noodles and crackers
Vegetables	Green, leafy, and yellow vegetables and other vegetables
Dried beans, nuts, and seeds	Beans, nuts, peas, seeds, etc.
Fruits	Vitamin C-rich fruits and other fruits
Meat, fish, and poultry	Fish and fish products, meat and meat products, poultry
Milk and milk products	Whole milk and milk products
Sugary sweetened beverages	Soft drinks, flavored juice drinks, energy drinks, etc.
Fats and oils	Coconut oil, palm oil, animal fat, butter, etc.

**Table 2:** Categories of food insecurity^1^.

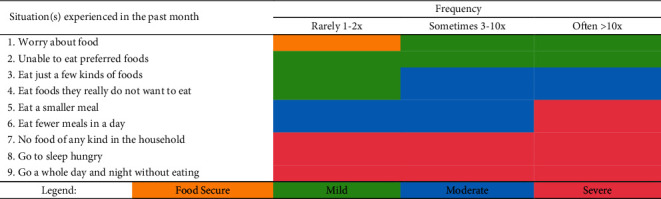

^1^Household Food Insecurity Access Scale Indicator Guide, V.3.

**Table 3:** FCS of the standard food group and current standard weights by the World Food Programme (WFP)^1^.

	Food items (examples)	Food groups (definitive)	Weight (definitive)
1	Rice, bread, noodles, biscuits, cookies, or any rice and cereal products like biko, suman (malagkit), puto, noodles/pasta, porridge (arrozcaldo/champorado), and others Cassava, potatoes and sweet potatoes, other tubers, and plantains	Main staples	2
2	Beans, peas, lentils, nuts, seeds, or foods made from these like pork and beans, guisantes de lata, and others	Pulses	3
3	Vegetables, leaves	Vegetables	1
4	Fruits	Fruits	1
5	Beef, goat, poultry, eggs, fish, and shellfish	Meat and fish	4
6	Milk, yogurt, and other dairy products	Milk	4
7	Sugar and sugar products, honey	Sugar	0.5
8	Oils, fats, and butter	Oil	0.5
9	Spices, tea, coffee, salt, fish powder, small amounts of milk for tea	Condiments	0

^1^World Food Programme (WFP) - Food Consumption Score (FCS) indicator.

**Table 4:** World food programme food consumption score^1^.

Score	Food consumption group
0–28	Poor food consumption
>28 to 42	Borderline food consumption
>42	Acceptable food consumption

^1^World Food Programme (WFP) Food Consumption Score (FCS) indicator.

**Table 5:** Household characteristics.

Characteristics (*n* = 9668)		All	Food Security				*p* value
			Secure	Mildly insecure	Moderately insecure	Severely insecure	
		*n* (%)	*n* (%)	*n* (%)	*n* (%)	*n* (%)	
	Sample, *n*	9668 (100)	3142 (32.5)	1253 (13)	3145 (32.5)	2128 (22)	
Household size	≤5 members	6163 (63.4)	2243 (71.4)	819 (65.2)	1895 (59.6)	1206 (56)	<0.001^*∗*^
	>5 members	3505 (36.6)	899 (28.6)	434 (34.8)	1250 (40.4)	922 (43.9)	

Place of residence	Rural	5948 (55)	1677 (47.6)	773 (54.5)	2116 (61.3)	1382 (57.3	<0.001^*∗*^
	Urban	3720 (45)	1465 (52.4)	480 (45.5)	1029 (32.7)	746 (35.1)	

Sex of household head	Male	7728 (79.3)	2429 (76.5)	1006 (79.4	2566 (81.5)	1727 (79.3)	<0.001^*∗*^
	Female	1940 (20.7)	719 (23.5)	247 (20.6)	579 (18.5)	407 (20.7)	

Wealth quintile	Poorest	2175 (20)	249 (6.6)	207 (14.5)	880 (25.8)	839 (35.4)	<0.001^*∗*^
	Poor	2081 (19.8)	387 (10.8)	262 (19.1)	861 (25.4)	571 (25.6)	
	Middle	1984 (20.5)	520 (15.5)	321 (24.9)	748 (24.6)	395 (19.9)	
	Rich	1842 (20.9)	830 (27.1)	285 (25.3)	491 (17.6)	236 (13.6)	
	Richest	1584 (18.7)	1153 (40)	178 (16.2)	165 (6.6)	85 (5.5)	

Educational level of the household head	No education	316 (3.1)	52 (1.6)	21 (1.5)	102 (3.2)	141 (6.1)	<0.001^*∗*^
	Elementary level	4008 (39.2)	903 (27.2)	484 (35.8)	1488 (45)	1133 (50.7)	
	High school level	3284 (35.2)	1028 (33.1)	471 (39.3)	1138 (37.9)	647 (32)	
	Vocational level	555 (6.2)	267 (9)	77 (6.6)	143 (3.8)	68 (3.8)	
	College level	1504 (16.3)	892 (29)	199 (16.7)	274 (9)	139 (7.4)	

Occupation of the household head	No occupation	660 (6.3)	118 (3.3)	68 (5)	246 (7.2)	228 (10.2)	<0.001^*∗*^
	Low-income occupation	4043 (38.3)	905 (25.6)	500 (36.6)	1547 (46.3)	1091 (46.8)	
	Middle-income occupation	1998 (21.9)	622 (20.7)	307 (25.3)	655 (22.5)	414 (21.1)	
	High-income occupation	2967 (33.5)	1497 (50.4)	378 (33.1)	697 (23.9)	395 (21.8)	

Electricity in the household	No (no connection/no electricity in the area)	982 (9.5)	95 (2.8)	99 (7.3)	370 (11.4)	418 (18.1)	<0.001^*∗*^
	Yes	8686 (90.5)	3047 (97.2)	1154 (92.7)	88.6)	1710 (81.9)	

Type of toilet facility	No toilet/not water-sealed	1455 (14.1)	200 (5.7)	132 (10.1)	526 (16.5)	597 (27.1)	<0.001^*∗*^
	Water-sealed (with/without flush)	8059 (85.6)	2892 (94.3)	1109 (89.9)	2571 (83.5)	1487 (72.9)	

Chi-square test; significant at *α* = 0.05; ^*∗*^significant.

**Table 6:** Distribution of household total food consumption by the food insecurity level.

Food groups		All	Food security				*p* value^*ψ*^
			Secure	Mild	Moderate	Severe	
Cereals and cereal products (g)	Mean	1507.6	1355.8	1503.9	1590.4	1611.3	<0.001^*∗*^^abcde^
	Standard deviation	920.1	886.8	880.1	890.8	1001.1	
	25th percentile	868.6	747.6	898.0	972.6	919.7	
	Median	1343.4	1180.5	1359.5	1445.2	1467.7	
	95th percentile	3140.2	2935.4	3100.3	3199.1	3338.1	

Starchy roots and tubers (g)	Mean	58.9	55.9	42.4	58.4	73.8	0.024^*∗*^^e^
	Standard deviation	297.9	260.8	203.2	295.3	386.4	
	25th percentile	0.0	0.0	0.0	0.0	0.0	
	Median	0.0	0.0	0.0	0.0	0.0	
	95th percentile	293.6	294.8	236.9	305.2	326.8	

Rice and rice products (g)	Mean	1303.5	1192.5	1302.5	1357.1	1388.7	<0.001^*∗*^^abce^
	Standard deviation	903.3	854.4	864.0	894.0	989.8	
	25th percentile	691.8	627.9	724.3	743.2	726.5	
	Median	1149.9	1031.9	1163.2	1253.4	1244.4	
	95th percentile	2915.9	2674.6	2771.9	2974.7	3137.2	

Vegetables (g)	Mean	528.3	484.7	528.3	560.8	544.7	<0.001^*∗*^^bc^
	Standard deviation	714.9	541.3	620.1	801.7	841.9	
	25th percentile	83.0	92.6	91.5	87.0	51.5	
	Median	336.6	319.9	347.8	370.4	303.3	
	95th percentile	1673.7	1538.0	1646.2	1785.0	1861.7	

Fruits (g)	Mean	145.6	170.9	145.6	134.2	124.8	0.016^*∗*^^c^
	Standard deviation	569.2	405.9	489.1	713.6	578.1	
	25th percentile	0.0	0.0	0.0	0.0	0.0	
	Median	0.0	0.0	0.0	0.0	0.0	
	95th percentile	837.0	954.8	834.5	775.0	689.4	

Meat, fish, and poultry (g)	Mean	707.7	849.1	750.9	637.4	577.6	<0.001^*∗*^^abcdef^
	Standard deviation	671.6	715.8	772.5	583.7	619.1	
	25th percentile	250.0	351.4	250.0	222.9	175.2	
	Median	549.7	679.4	567.6	500.0	436.6	
	95th percentile	1928.4	2138.9	2038.9	1744.6	1565.6	

Dried beans, nuts, and seeds (g)	Mean	34.0	33.6	38.9	34.8	30.5	0.031^*∗*^^e^
	Standard deviation	82.0	82.6	84.6	83.2	77.6	
	25th percentile	0.0	0.0	0.0	0.0	0.0	
	Median	0.0	0.0	0.0	0.0	0.0	
	95th percentile	173.5	159.4	197.1	180.3	160.3	

Milk and milk products (g)	Mean	157.9	198.1	184.1	132.0	121.4	<0.001^*∗*^^bcde^
	Standard deviation	498.2	466.2	510.6	516.0	505.0	
	25th percentile	0.0	0.0	0.0	0.0	0.0	
	Median	0.0	0.0	0.0	0.0	0.0	
	95th percentile	792.2	962.3	850.0	674.8	651.2	

Sugary sweetened beverages (g)	Mean	60.9	68.1	65.9	57.7	52.1	0.003^*∗*^^c^
	Standard deviation	166.4	172.7	232.3	156.8	115.9	
	25th percentile	2.8	2.6	2.9	3.1	2.8	
	Median	22.0	22.0	22.0	22.5	20.0	
	95th percentile	266.8	306.0	257.8	252.0	250.0	

Fats and oils (g)	Mean	58.3	65.3	59.4	55.8	50.9	<0.001^*∗*^^bc^
	Standard deviation	119.9	136.6	112.7	119.9	94.3	
	25th percentile	7.2	9.9	11.2	7.4	0.0	
	Median	31.4	34.7	36.2	30.7	25.5	
	95th percentile	183.0	198.0	174.2	177.6	175.3	

^*ψ*^One-way analysis of variance with multiple comparison using Bonferroni adjustment, *α* = 0.05, ^*∗*^significant; ^NS^not significant. ^a^Comparing food secure to mild food insecure household, ^b^comparing food secure to moderate food insecure household, ^c^comparing food secure to severely food insecure household, ^d^comparing mild food insecure to moderate food insecure household, ^e^comparing mild food insecure to severely food insecure household, and ^f^comparing moderate food insecure to severely food insecure household.

**Table 7:** Distribution of household's total energy and nutrient intakes by the food insecurity level.

Nutrients		All	Food security				*p* value^*ψ*^
			Secure	Mild	Moderate	Severe	
Total energy (kcal)	Mean	7607.2	7383.4	7664.7	7784.7	7641.3	0.001^*∗*^^b^
	Standard deviation	4093.0	4182.2	3955.9	3977.4	4194.2	
	25th percentile	4744.5	4485.5	4891.1	5040.4	4707.6	
	Median	6994.8	6638.0	7077.0	7224.2	7021.1	
	95th percentile	15181.7	15352.4	14993.0	15151.0	15219.3	

Total carbohydrates (g)	Mean	1340.4	1220.5	1338.6	1407.9	1418.8	<0.001^*∗*^^abcde^
	Standard deviation	750.3	721.3	716.4	743.1	798.6	
	25th percentile	806.7	708.0	832.2	892.3	835.2	
	Median	1213.5	1079.2	1217.1	1286.0	1304.1	
	95th percentile	2732.8	2607.9	2605.9	2769.9	2928.0	

Total protein (g)	Mean	228.2	238.0	234.1	224.8	215.3	<0.001^*∗*^^bce^
	Standard deviation	129.8	140.8	134.1	119.1	124.5	
	25th percentile	138.3	140.4	138.9	142.6	129.1	
	Median	204.7	211.1	207.1	203.9	196.9	
	95th percentile	471.4	509.4	485.9	453.9	442.1	

Total fat (g)	Mean	145.6	168.4	152.8	137.4	119.8	<0.001^*∗*^^abcdef^
	Standard deviation	130.6	140.0	129.9	126.4	116.1	
	25th percentile	55.8	71.2	64.0	52.3	43.3	
	Median	108.5	130.8	122.1	100.7	85.7	
	95th percentile	397.9	437.7	395.4	384.3	342.9	

Calcium (g)	Mean	38.5	38.9	39.3	38.8	37.1	0.011^*∗*^^ce^
	Standard deviation	23.1	24.4	22.6	22.5	22.3	
	25th percentile	22.7	21.9	23.7	23.8	21.5	
	Median	34.2	34.2	35.1	34.8	33.3	
	95th percentile	82.1	84.8	83.9	81.1	78.4	

Iron (mg)	Mean	1649.9	1627.8	1710.2	1659.1	1633.1	0.223^NS^
	Standard deviation	1261.2	1262.1	1354.9	1191.5	1301.6	
	25th percentile	878.6	848.7	912.0	908.5	855.1	
	Median	1341.7	1313.7	1373.8	1376.5	1323.9	
	95th percentile	3904.4	3917.2	4061.3	3803.0	3866.3	

Vitamin A (*µ*g)	Mean	1649.5	1930.2	1699.4	1536.0	1373.2	<0.001^*∗*^^bce^
	Standard deviation	2779.4	3262.5	2840.7	2427.0	2392.1	
	25th percentile	519.8	584.9	576.5	526.1	420.6	
	Median	984.3	1073.1	1040.8	977.6	833.7	
	95th percentile	4474.1	6182.7	4838.4	4102.4	3566.3	

Thiamin (mg)	Mean	3.4	3.5	3.4	3.3	3.2	<0.001^*∗*^^c^
	Standard deviation	2.4	2.6	2.4	2.4	2.3	
	25th percentile	1.8	1.7	1.9	1.8	1.7	
	Median	2.8	2.8	2.9	2.8	2.7	
	95th percentile	7.9	8.5	8.1	7.4	7.3	

Riboflavin (mg)	Mean	2.8	3.0	2.9	2.7	2.6	<0.001^*∗*^^bcde^
	Standard deviation	2.0	2.1	2.0	1.8	1.9	
	25th percentile	1.5	1.6	1.6	1.6	1.4	
	Median	2.4	2.5	2.5	2.3	2.2	
	95th percentile	6.5	7.3	6.6	5.9	5.7	

Niacin (mg)	Mean	75.8	78.4	76.8	74.9	73.0	<0.001^*∗*^^bc^
	Standard deviation	45.8	49.1	45.9	43.0	44.5	
	25th percentile	43.9	44.9	44.5	44.7	41.6	
	Median	67.1	68.0	67.2	67.6	65.0	
	95th percentile	160.5	171.2	158.5	155.4	155.1	

Vitamin C (mg)	Mean	182.1	180.2	179.6	186.2	180.2	0.611^NS^
	Standard deviation	209.3	197.4	201.6	213.9	223.6	
	25th percentile	41.9	46.3	47.2	42.6	32.1	
	Median	121.5	122.8	121.6	125.2	112.2	
	95th percentile	587.0	562.0	559.8	616.9	613.9	

^*ψ*^One-way analysis of variance with multiple comparison using Bonferroni adjustment, *α* = 0.05, ^*∗*^significant; ^NS^not significant. ^a^Comparing food secure to mild food insecure household, ^b^comparing food secure to moderate food insecure household, ^c^comparing food secure to severely food insecure household, ^d^comparing mild food insecure to moderate food insecure household, ^e^comparing mild food insecure to severely food insecure household, and ^f^comparing moderate food insecure to severely food insecure household.

**Table 8:** Household food security by household food consumption classification.

Food security	Food consumption classification		
	Poor	Borderline	Acceptable
	*N* (%)	*N* (%)	*N* (%)
Food secure	48 (16)	257 (21)	2837 (36)
Mildly food insecure	27 (9)	122 (10)	1104 (14)
Moderately food insecure	83 (26)	449 (35)	2613 (31)
Severely food insecure	152 (49)	424 (34)	1552 (19)

**Table 9:** Percentage contribution of each food group to the household total energy intake by food security.

Food groups	Overall (%)	Food security			
		Food secure (%)	Mildly food insecure (%)	Moderately food insecure (%)	Severely food insecure (%)
Rice	68	63	67	71	74
Meat	14	18	14	12	10
Fats and oils	7	8	7	7	6
Sugar sweetened beverages	2	2	2	2	2
Vegetables	2	2	3	3	2
Milk	1	2	2	1	1
Fruits	1	2	1	1	1
Eggs	1	2	2	1	1
Dried beans, and nuts and peas	1	1	1	1	1
Starchy roots and tubers	1	1	1	1	1

**Table 10:** Relationship between dietary consumption and food insecurity level (*n* = 9668).

Dietary outcome variables	Food secure	Food security			*R* ^2^ (%)
		Mildly food insecure	Moderately food insecure	Severely food insecure	
		*B* (95% CI)	*B* (95% CI)	*B* (95% CI)	
*Food consumption score*					
Model 1^a^	ref	−2.59 (−3.82, −1.36)^*∗∗*^	−7.1 (−8, −6.14)^*∗∗*^	−12 (−13.1, −10.95)^*∗∗*^	10
Model 2^b^	ref	0.68 (−0.52, 1.9)^NS^	−1.46 (−2.45, −0.47)^*∗*^	−4.45 (−5.58, −3.32)^*∗∗*^	18.6

*Energy (kcal)*					
Model 1^a^	ref	−133.94 (−344.62, 76.74)^NS^	−277.1 (−445.33, −109.84)^*∗*^	−632.47 (−818.25, −446.69)^*∗∗*^	42.3
Model 2^b^	ref	18.1 (−195.5, 231.67)^NS^	−40.19 (−219.13, 138.74)^NS^	−331.3 (−535.1, −127.59^*∗*^	43.1

*Protein (g)*					
Model 1^a^	ref	−14.22 (−21.74, −6.7)^*∗∗*^	−29.48 (−35.1, −23.87)^*∗∗*^	−44.83 (−51, −38.61)	35.1
Model 2^b^	ref	−0.96 (−8.50, 6.57)^NS^	−8 (−13.85, −2.17)^*∗*^	−18.47 (−25.1, −11.85)^*∗∗*^	37.5

*Total carbohydrates (g)*					
Model 1^a^	ref	31.6 (−4.84, 68)^NS^	45.41 (15.94, 74.88)^*∗*^	18.75 (−13.65, 51.15)^NS^	43.8
Model 2^b^	ref	8.84 (−28.24, 45.92)^NS^	1.35 (−29.99, 32.7)^NS^	−35.88 (−71.82, 0.06)^NS^	44.8

*Total fats (g)*					
Model 1^a^	ref	−19.52 (−28.1, −11)^*∗∗*^	−34.98 (−41.72, −28.24)^*∗∗*^	−56.65 (−63.86, −49.43)^*∗∗*^	17.9
Model 2^b^	ref	1.41 (−6.96, 9.79)^NS^	0.98 (−6.1, 8.1)^NS^	−11 (−18.75, −3.26)^*∗*^	24.7

*Calcium (mg)*					
Model 1^a^	ref	−1.45 (−2.78, −0.12)^*∗*^	−2.96 (−4, −1.92)^*∗∗*^	−5.81 (−6.95, −4.66)^*∗∗*^	29.1
Model 2^b^	ref	0.62 (−0.72, 1.95)^NS^	0.41 (−0.71, 1.53)^NS^	−1.52 (−2.74, 2.1)^*∗*^	31.3

*Iron (mg)*					
Model 1^a^	ref	−3.82 (−82.95, 75.30)^NS^	−100 (−157.3, −42.72)^*∗*^	−186.64 (−251, −122.28)^*∗∗*^	17.3
Model 2^b^	ref	47 (−33.75, 127.82)^NS^	−11.85 (−74.76, 51.1)^NS^	−72.91 (142.51, −3.31)^*∗*^	18.1

*Vitamin A RE (µg)*					
Model 1^a^	ref	−234.6 (−448.69, −20.54)^*∗*^	−432.89 (−583.33, −282.44)^*∗∗*^	−619.5 (−794.85, −444.16)^*∗∗*^	3.1
Model 2^b^	ref	21.39 (−199.84, 242.62)^NS^	−13.42 (−177.19, 150.35)^NS^	−113.13 (−307.58, 81.32)^NS^	5

*Thiamin (mg)*					
Model 1^a^	ref	−0.20 (−0.35, −0.05)^*∗*^	−0.38 (−0.5,−0.26)^*∗∗*^	−0.62 (−0.74, −0.49)^*∗∗*^	22.2
Model 2^b^	ref	−0.01 (−0.16, 0.15)^NS^	−0.06 (−0.19, 0.06)^NS^	−0.21 (−0.35, −0.07)^*∗*^	24.2

*Riboflavin (mg)*					
Model 1^a^	ref	−0.15 (−0.28, −0.02)^*∗*^	−0.45 (−0.54, −0.35)^*∗∗*^	−0.65 (−0.76, −0.54)^*∗∗*^	21
Model 2^b^	ref	0.09 (−0.04, 0.22)^NS^	−0.04 (−0.14, 0.06)^NS^	−0.14 (−0.25, −0.02)^*∗*^	25

*Niacin (mg)*					
Model 1^a^	ref	−4.86 (−7.53, −2.19)^*∗∗*^	−9 (−11, −7.19)^*∗∗*^	−12.8 (−15, −10.6)	31.5
Model 2^b^	ref	−1.15 (−3.84, 1.53)^NS^	−3.12 (−5.21, −1)^*∗*^	−5.89 (−8.28, −3.5)^*∗∗*^	33.2

*Vitamin C (mg)*					
Model 1^a^	ref	−14.1 (−26.66, −1.46)^*∗*^	−11.38 (−21.55, −1.2)^*∗*^	−25.75 (−3, −14.5)^*∗∗*^	7.4
Model 2^b^	ref	−5.69 (−18.54, 7.16)^NS^	−0.82 (−11.65, 10)^NS^	−18.2 (−30.46, 6)^*∗*^	8.5

^*∗∗*^ *p* value <0.001, ^*∗*^*p* value <0.05, ^NS^not significant, ^a^adjusted for household size, place of residence, and sex of the household head only, ^b^adjusted for household size, place of residence, sex of the household head, electricity of the household, and type of toilet facility of the household, educational level of the household head, occupational level of household head, and socio-economic status of the household (wealth quintile).

**Table 11:** Association between dietary inadequacy and food insecurity level (*n* = 9668).

Dietary	Food security				Pseudo-*R*^2^
	Food secure	Mildly food insecure	Moderately food insecure	Severely food insecure	
		OR (95% CI)	OR (95% CI)	OR (95% CI)	
*Poor/borderline food consumption (FCS < 42%)*					
Model 1^a^	Ref	1.29 (1, 1.6)^*∗*^	1.91 (1.63, 2.24)^*∗∗*^	3.36 (3.1, 4.25)^*∗∗*^	6.3
Model 2^b^	Ref	0.9 (0.72, 1.13)^NS^	1.1 (0.9, 1.28)^NS^	1.69 (1.41, 2)^*∗∗*^	11.4

*Prevalence of inadequacy of energy*					
Model 1^a^	Ref	1.20 (1.03, 1.4)^*∗*^	1.25 (1.12, 1.37)^*∗∗*^	1.57 (1.37, 179)^*∗∗*^	3.6
Model 2^b^	Ref	1.13 (0.97, 1.33)^NS^	1.15 (1, 1.30)^*∗*^	1.45 (1.24, 1.67)^*∗∗*^	3.9

*Prevalence of inadequacy of protein*					
Model 1^a^	Ref	1.33 (1.15, 1.54)^*∗∗*^	1.61 (1.45, 1.80)^*∗∗*^	2.1 (1.83, 2.33)^*∗∗*^	2.3
Model 2^b^	Ref	1.11 (0.96, 1.29)^NS^	1.2 (1, 1.34)^*∗*^	1.43 (1.25, 1.64)^*∗∗*^	4

*Prevalence of inadequacy of calcium*					
Model 1^a^	Ref	1.15 (0.96, 1.38)^NS^	1.24 (1.1, 1.42)^*∗*^	1.45 (1.23,1.71)^*∗∗*^	1.7
Model 2^b^	Ref	1.1 (0.89, 1.31)^NS^	1.11 (0.95, 1.29)^NS^	1.26 (1.1, 1.52)^*∗*^	

*Prevalence of inadequacy of iron*					
Model 1^a^	Ref	1.21 (0.96, 1.53)^NS^	1.37 (1.14, 1.64)^*∗*^	1.43 (1.16, 1.77)^*∗*^	4.5
Model 2^b^	Ref	1.1 (0.86, 1.4)^NS^	1.17 (0.95, 1.43)^NS^	1.2 (0.94, 1.54)^NS^	4.9

*Prevalence of inadequacy of vitamin A RE*					
Model 1^a^	Ref	1.19 (1, 1.4)^*∗*^	1.6 (1.41, 1.81)^*∗∗*^	2.1 (1.78, 2.38)^*∗∗*^	2.9
Model 2^b^	Ref	0.99 (0.84, 1.17)^NS^	1.16 (1, 1.34)^*∗*^	1.38 (1.18, 1.63)^*∗∗*^	4.5

*Prevalence of inadequacy of thiamin*					
Model 1^a^	Ref	1.23 (1.1, 1.42)^*∗*^	1.56 (1.39, 1.75)^*∗∗*^	1.64 (1.45, 1.87)^*∗∗*^	1.9
Model 2^b^	Ref	1.1 (0.9, 1.22)^NS^	1.19 (1.1, 1.35)^*∗*^	1.18 (1, 1.36)^*∗*^	3.2

*Prevalence of inadequacy of riboflavin*					
Model 1^a^	Ref	1.35 (1.14, 1.61)^*∗∗*^	1.91 (1.67, 2.19)^*∗∗*^	2.14 (1.82, 2.51)^*∗∗*^	3.8
Model 2^b^	Ref	1.1 (0.92, 1.31)^NS^	1.34 (1.16, 1.56)^*∗∗*^	1.36 (1.14, 1.63)^*∗*^	5.7

*Prevalence of inadequacy of niacin*					
Model 1^a^	Ref	1.37 (1.11, 1.69)^*∗*^	1.70 (1.45, 2)^*∗∗*^	2.15 (1.82, 2.54)^*∗∗*^	2.2
Model 2^b^	Ref	1.11 (0.89, 1.37)^NS^	1.2 (1, 1.43)^*∗*^	1.35 (1.12, 1.63)^*∗*^	

*Prevalence of inadequacy of vitamin C*					
Model 1^a^	Ref	1.15 (0.99, 1.33)^NS^	1.18 (1.1, 1.32)^*∗*^	1.33 (1.17, 1.5)^*∗∗*^	0.7
Model 2^b^	Ref	1.1 (0.91, 1.24)^NS^	1.1 (0.97, 1.24)^NS^	1.28 (1.1, 1.47)^*∗*^	1.5

^*∗∗*^ *p* value <0.001, ^*∗*^*p* value <0.05, ^NS^not significant. ^a^Adjusted for household size, place of residence, and sex of the household head only. ^b^Adjusted for household size, place of residence, sex of the household head, electricity of the household, and type of toilet facility of the household, educational level of the household head, occupational level of household head, and socioeconomic status of the household (wealth quintile).

## Data Availability

The dataset used for this study can be requested via an online application from the Department of Science and Technology, Food and Nutrition Research Institute's official website (http://enutrition.fnri.dost.gov.ph/site/puf-preview.php?xx=201596).
